# Unexpected Accumulation of ncm^5^U and ncm^5^s^2^U in a *trm9* Mutant Suggests an Additional Step in the Synthesis of mcm^5^U and mcm^5^s^2^U

**DOI:** 10.1371/journal.pone.0020783

**Published:** 2011-06-07

**Authors:** Changchun Chen, Bo Huang, James T. Anderson, Anders S. Byström

**Affiliations:** 1 Department of Molecular Biology, Umeå University, Umeå, Sweden; 2 Division of Epidemiology, Department of Medicine and Public Health, Vanderbilt University School of Medicine, Nashville, Tennessee, United States of America; 3 Department of Biological Sciences, Marquette University, Milwaukee, Wisconsin, United States of America; Max-Planck-Institute for Terrestrial Microbiology, Germany

## Abstract

**Background:**

Transfer RNAs are synthesized as a primary transcript that is processed to produce a mature tRNA. As part of the maturation process, a subset of the nucleosides are modified. Modifications in the anticodon region often modulate the decoding ability of the tRNA. At position 34, the majority of yeast cytosolic tRNA species that have a uridine are modified to 5-carbamoylmethyluridine (ncm^5^U), 5-carbamoylmethyl-2′-O-methyluridine (ncm^5^Um), 5-methoxycarbonylmethyl-uridine (mcm^5^U) or 5-methoxycarbonylmethyl-2-thiouridine (mcm^5^s^2^U). The formation of mcm^5^ and ncm^5^ side chains involves a complex pathway, where the last step in formation of mcm^5^ is a methyl esterification of cm^5^ dependent on the Trm9 and Trm112 proteins.

**Methodology and Principal Findings:**

Both Trm9 and Trm112 are required for the last step in formation of mcm^5^ side chains at wobble uridines. By co-expressing a histidine-tagged Trm9p together with a native Trm112p in *E. coli*, these two proteins purified as a complex. The presence of Trm112p dramatically improves the methyltransferase activity of Trm9p *in vitro*. Single tRNA species that normally contain mcm^5^U or mcm^5^s^2^U nucleosides were isolated from *trm9*Δ or *trm112*Δ mutants and the presence of modified nucleosides was analyzed by HPLC. In both mutants, mcm^5^U and mcm^5^s^2^U nucleosides are absent in tRNAs and the major intermediates accumulating were ncm^5^U and ncm^5^s^2^U, not the expected cm^5^U and cm^5^s^2^U.

**Conclusions:**

Trm9p and Trm112p function together at the final step in formation of mcm^5^U in tRNA by using the intermediate cm^5^U as a substrate. In tRNA isolated from *trm9*Δ and *trm112*Δ strains, ncm^5^U and ncm^5^s^2^U nucleosides accumulate, questioning the order of nucleoside intermediate formation of the mcm^5^ side chain. We propose two alternative explanations for this observation. One is that the intermediate cm^5^U is generated from ncm^5^U by a yet unknown mechanism and the other is that cm^5^U is formed before ncm^5^U and mcm^5^U.

## Introduction

Transfer RNAs are adapter molecules, which decode mRNA into protein and thereby play a central role in gene expression. The primary tRNA transcript is processed by different endo and exonucleases, and tRNA modifying enzymes to produce a mature tRNA [1], [2], [3]. In this maturation process, a subset of the four normal nucleosides adenosine (A), guanosine (G), cytidine (C) and uridine (U) are modified [2], [3]. The modifications are introduced post-transcriptionally, and the formation of a modified nucleoside may require one or several enzymatic steps [2], [3]. Of the 50 modified nucleosides so far identified in eukaryotic tRNAs, 25 are present in cytoplasmic tRNAs from *S. cerevisiae* [2], [4], [5]. In the anticodon region, especially in positions 34 (wobble position) and 37, nucleosides are frequently modified. Modified nucleosides in these positions are important for reading frame maintenance and efficient decoding during translation [2], [3]. In yeast, there are in total 42 cytosolic tRNA species, of which 11 have a uridine at position 34 modified to 5-carbamoylmethyluridine (ncm^5^U), 5-carbamoylmethyl-2′-O-methyluridine (ncm^5^Um), 5-methoxycarbonylmethyl-uridine (mcm^5^U) or 5-methoxycarbonylmethyl-2-thiouridine (mcm^5^s^2^U) [6]. The formation of these nucleosides requires addition of mcm or ncm side chains at the 5-position of the uracil moity and a subset of these tRNAs also have a thio (s^2^) group at the 2-position of U_34_ or a methylation at the 2′ position of the ribose.

**Figure 1:**
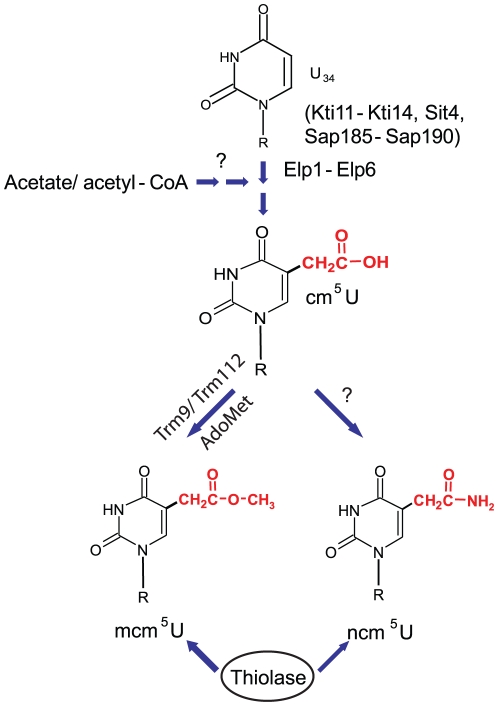
Model for formation of mcm^5^ side chain at wobble uridines. The Elongator complex (Elp1-Elp6) and its potential regulators are required for the formation of cm^5^U. A methyl group is added to cm^5^U by Trm9p/Trm112p complex in tRNA species that in their mature form should have a mcm^5^ side chain. The cm^5^U in other tRNA species are converted to ncm^5^U by an unknown enzyme. For tRNAs that should contain a s^2^ group, presence of a mcm^5^ or ncm^5^ side chain is a prerequisite for efficient thiolation.

The common step in synthesis of ncm^5^ and mcm^5^ side chains at U_34_ in tRNAs requires at least 11 gene products (Figure 1). Deletion strains missing one of *ELP1-ELP6*, *KTI11*, *KTI12*, *KTI14* or *SIT4* genes, or both *SAP185and SAP190* genes completely lack the mcm^5^U, mcm^5^s^2^U and ncm^5^U nucleosides, whereas a *kti13* deletion mutant show dramatically reduced levels of these nucleosides [7], [8]. In strains with these genes mutated, no intermediates of mcm^5^U or ncm^5^U have been detected, whereas s^2^U is detected in tRNAs normally containing mcm^5^s^2^U [7], [8], [9], [10], [11], [12]. Thus, these gene products are required for an early step in synthesis of mcm^5^ and ncm^5^ groups (Figure 1). The earliest intermediate in the synthesis of mcm^5^U and ncm^5^U that has been detected is cm^5^U, and there is evidence that it originates from a metabolite related to acetyl-CoA [13] (Figure 1).

The *ELP1-ELP6* gene products form the Elongator complex that consists of a core complex Elp1-Elp3 and a sub complex Elp4-Elp6 [14], [15], [16]. In the C-terminal part of Elp3p there is a potential acetyl-CoA binding domain [17], and the central region shares homology to the Radical SAM superfamily [18]. Members of this family contain an iron-sulphur (FeS) cluster and use S-adenosylmethionine (SAM) to catalyze a variety of radical reactions. The presence of a FeS cluster and ability to bind SAM has been verified for the *M. jannaschii* Elp3p homologue [18], whereas no binding of SAM to *S. cerevisiae* Elongator complex was observed [19]. At least Elp1 and Elp3 of Elongator core complex are in intimate contact with tRNA that is modified with a mcm side chain at U_34_ [7]. The *KTI11-KTI14*, *SIT4* or *SAP185 SAP190* gene products seem to regulate the activity of Elongator complex [20], [21], [22], [23], [24], [25], [26], [27], [28], [29], [30].

The last step in formation of mcm^5^ side chain of U_34_ is a methyl esterification of cm^5^ [13], and requires Trm9p/Trm112p in yeast and ALKBH8/TRM112 in mammalians [31], [32], [33]. We confirm that Trm112p is also required for the last step of mcm^5^ side chain formation at position 34 in a subset of tRNAs. *In vivo*, Trm112p is essential for the methyl esterification to mcm^5^U_34_, and *in vitro* Trm112p improves the methyltransferase activity of Trm9p. The observation that the major intermediates accumulating in *trm9* and *trm112* mutants are ncm^5^U and ncm^5^s^2^U and not the expected cm^5^U and cm^5^s^2^U raises the question; what is the order of intermediates formed in biosynthesis of the mcm^5^ side chain of U_34_?

## Materials and Methods

### Yeast strains, media and genetic procedures

Strains used in this report, except those from the yeast deletion collection (Open Biosystems), are listed in Table S1A. Yeast media, genetic procedures and yeast transformation have been described previously [34]. To construct *mtq2::KanMX6* and *trm112::KanMX6* deletions, oligonucleotides (2104 and 2015, 1391 and 1392) in Table S1B containing 45nt sequence homology flanking the *MTQ2* and *TRM112* genes were used to amplify the *KanMX6* cassette [35]. To delete *TRM9*, *TRM11* and *LYS9* in W303 strains, chromosomal DNA from the corresponding null mutants in the yeast deletion collection (Open Biosystems) were used as templates. The *KanMX6* cassette together with 300–500 base pair flanking sequences to each gene were amplified with specific primers (1035 and 1036 for *TRM9*, 1950 and 1951 for *TRM11*, and 2059 and 2060 for *LYS9*) listed in Table S1B. The PCR products were introduced into diploid yeast strain UMY3104 and transformants were selected on YEPD plates containing 200 µg/ml Geneticin (G418). Transformants were sporulated and tetrad analysis verified a 2∶2 segregation of mating type and G418 resistance. Deletions were confirmed by PCR. The double mutants *trm9*Δ *trm11*Δ, *trm9*Δ *lys9*Δ, *trm9*Δ *mtq2*Δ, *trm11*Δ *lys9*Δ, *trm11*Δ *mtq2*Δ and *lys9*Δ *mtq2*Δ were generated by crossing the single mutants. The quadruple mutant was generated in a cross between *trm9*Δ *lys9*Δ and *trm11*Δ *mtq2*Δ.

### Plasmid constructions

To generate the expression vector for the Trm9 protein, *TRM9* gene was amplified by PCR using oligos 2015 and 2016 (Table S1B) and W303-1A genomic DNA as template. The PCR product was digested with *Bam*H1 and *Hind*III, and subcloned to the corresponding sites of the expression vector pRSF-Duet1 (Novagen), generating an in frame fusion with the histidine tag. To construct the Trm9p-Trm112p co-expression vector, the *TRM112* gene was amplified from W303-1A genomic DNA using oligos 2013 and 2014 (Table S1B) and cloned into the pRSF-Duet1-*TRM9* vector using *Nde*I and *Xho*I.

### Protein purification

The expression vectors were introduced into BL21(DE3)pLysS competent cells. Overnight cultures of transformed cells were grown in LB media containing 50 µg/ml Kanamycin at 37°C. Cultures were diluted to OD_600_ 0.05 and grown to OD_600_ 0.5 at 37°C. Cultures were placed on ice for 10 minutes. IPTG was added to a final concentration of 120 µg/ml and protein expression was induced at 15°C overnight. Harvested cell pellets were washed once by 0.9% NaCl and resuspended in breaking buffer (20 mM Tris pH 8.0, 10 mM imidazole, 150 mM NaCl, 0.2% NP-40, 2 mM β-mercaptoethanol) in the presence of proteinase inhibitor cocktail (Roche). Cells were broken by sonication and the cell extract was clarified by centrifugation at 16,000 g for 1 hour. The supernatant was mixed with TALON resin, equilibrated with breaking buffer and incubated at 4°C for 2 hours. The protein bound TALON resin was first washed with buffer 1 (20 mM Tris pH 8.0, 10 mM imidazole, 150 mM NaCl, 2 mM β-mercaptoethanol) and then with buffer 2 (20 mM Tris pH 8.0, 10 mM imidazole, 500 mM NaCl, 2 mM β-mercaptoethanol). Proteins were eluted with 330 mM imidazole and dialyzed overnight against storage buffer (25 mM Tris pH 8.0, 150 mM NaCl, 5 mM DTT, 10% glycerol) and kept at 4°C for future use.

### Methyltransferase reaction

In the methyltransferase reaction, 50 µl of 2X reaction buffer (200 mM Tris 7.5, 0.2 mM EDTA, 20 mM MgCl_2_, 20 mM NH_4_Cl) was mixed with 20 µl [^3^H]AdoMet (0.55 mCi/ml, Perkin Elmer) and 20 µg tRNA, incubated at 37°C for 5 minutes. The methyltransferase reaction was initiated by adding 10 µg Trm9p or Trm9p-Trm112p. Aliquots of the reaction was withdrawn at different time points and mixed with 1 ml of 5% ice cold trichloroacetic acid (TCA). The tubes were incubated on ice for 10 minutes and samples were vacuum filtered through nitrocellulose filter (Millipore 0.45 µm). The [^3^H] incorporation was measured using a Wallac 1409 scintillation counter. To analyze [^3^H] incorporation in total tRNA by HPLC, 200 µg of tRNA was used. After 30 minutes of methyltransferase reaction, 2.5 volume of 99% ice cold ethanol was added into the reaction and samples were centrifuged for 30 minutes in eppendorf tubes at maximum speed. The pellet was resuspended in MQ water, digested with nuclease P1 and analyzed by HPLC [36]. The [^3^H] incorporation was monitored by a flow scintillation analyzer (Packard Bioscience).

### Single tRNA isolation

Yeast cells were grown in 2L YEPD at 30°C to OD600 = 1.5. Total tRNA was prepared as described [36]. Single tRNA species were isolated from total tRNA by hybridizing to biotinylated complementary oligonucleotides [36] and separated from total tRNA by attachment to streptavidin coated Dynabeads M-280 (Invitrogen). The single tRNAs were digested to nucleosides with nuclease P1 followed by bacterial alkaline phosphatase (BAP) treatment [0.2 M (NH_4_)_2_SO_4_ pH 8.3], and analyzed by HPLC [37].

## Results and Discussion

### Trm112p is required for the methyl esterification of mcm^5^U and mcm^5^s^2^U

In a global analysis of protein complexes in yeast, Trm112p was found to interact with three methyltransferases Trm9p, Trm11p and Mtq2p [38], [39], [40], [41]. In addition, Trm112p interacts with the saccharopine dehydrogenase Lys9p, the essential DEAH-box ATP-dependent RNA helicase Ecm16p and an essential component of the RSC chromatin remodeling complex Sfh1p [38], [39], [40], [41]. The *N^2^*-Monomethylguanosine-10 (m^2^G_10_) methyltransferase Trm11p, as well as the eRF1 methyltranferase Mtq2p, has to be in complex with Trm112p to be active [42], [43]. Trm9p is required for the methyl esterification of modified uridine nucleosides, resulting in the formation of 5-methylcarbonylmethyluridine (mcm^5^U_34_) and 5-methylcarbonylmethyl-2-thiouridine (mcm^5^s^2^U_34_) present in a subset of tRNA species in yeast, including 
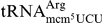
 and 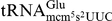 [31]. In the methyl esterification reaction of these tRNAs, cm^5^U_34_ and cm^5^s^2^U_34_ were suggested to be the substrates [13], [31], [32,].

**Figure 2:**
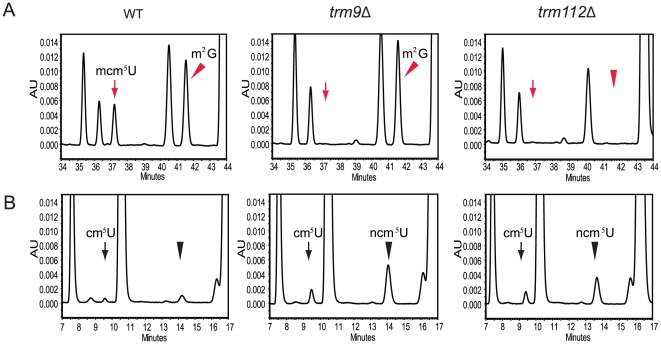
*trm9* and *trm112* mutants are lacking the mcm^5^ side-chain in 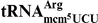 at wobble uridines. HPLC analysis of modified tRNA nucleosides from wild-type (UMY3169, left panels), *trm9::KanMX4* (Open Biosystems, middle panels) and *trm112::KanMX4* (UMY3330, right panels). Arrows in red and black indicate expected retention time of mcm^5^U and cm^5^U, respectively. Arrow heads in red and black indicate expected retention time of m^2^G and ncm^5^U, respectively. (A), Part of the chromatogram between retention times 34 and 44 min is shown. (B), Part of the chromatogram between retention times 7 and 17 min is shown. The small peak in wild-type at 14 min represents an unrelated compound with a spectrum different from ncm^5^U. The chromatograms were monitored at 254 nm.

**Figure 3:**
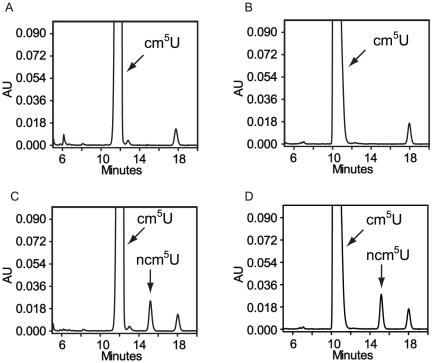
Nucleoside ncm^5^U is not generated by amidation of cm^5^U during conversion of tRNA into nucleosides. Synthetic cm^5^U (A and B) or a mixture of synthetic cm^5^U and ncm^5^U (C and D) were treated with nuclease P1 for 16 hours, followed by a 2 hours incubation with either water (A and C) or bacterial alkaline phosphatase (BAP) (B and D). Parts of the chromatogram of HPLC analysis between 5 and 20 min are shown. The chromatograms were monitored at 254 nm.

Both Trm9 and Trm112 are required for methyl esterification to mcm^5^U and mcm^5^s^2^U [31], [32], [33]. To analyze the tRNA modification status in these two mutants, total tRNA from *trm9*Δ, *trm112*Δ and wild type strains were isolated, digested to nucleosides and analyzed by HPLC. Similar to previous reports [31], [32], [33], total tRNA isolated from *trm9* and *trm112* deletion mutants lacked mcm^5^U and mcm^5^s^2^U nucleosides (data not shown). In order to provide a more detailed analysis of all possible nucleoside intermediates in *trm9*Δ and *trm112*Δ mutants, single tRNA species, 
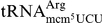
, 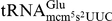 and 
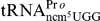
, were isolated from wild type, *trm9*Δ and *trm112*Δ strains and the purified tRNAs were digested to nucleosides and analyzed by HPLC (Figure 2, Table 1, data not shown). As expected, the ncm^5^U nucleoside was present in 
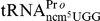
 independent if the tRNA was isolated from *trm9*Δ, *trm112*Δ or wild type strains (data not shown). The mcm^5^U and mcm^5^s^2^U nucleosides were present in 
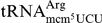
 and 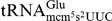 isolated from wild type but not from *trm9*Δ and *trm112*Δ cells (Figure 2, Table 1). In 
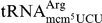
isolated from *trm9*Δ and *trm112*Δ strains, we observed the appearance of ncm^5^U and cm^5^U (Figure 2B, Table 1). Interestingly, the major intermediate of the mcm^5^U nucleoside generated in the *trm9*Δ and *trm112*Δ mutants is ncm^5^U (Figure 2, Table 1). The presence of ncm^5^U and cm^5^U has also been observed in 
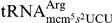
, 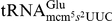 and 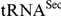 isolated from an *alkbh8* ^−/−^ mice [32], [44]. In 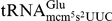 isolated from the *trm9*Δ and *trm112*Δ strains, there was a complete lack of mcm^5^s^2^U and a concomitant increase of cm^5^U, ncm^5^U and ncm^5^s^2^U (Table 1). The presence of cm^5^U supports the earlier observation that formation of a completed mcm^5^ side chain appears to be a prerequisite for efficient and complete thiolation of position 2 in mcm^5^s^2^U containing tRNAs [10], [11], [12], [32]. An unexpected observation was that the major species accumulating in 
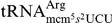
 and 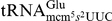 isolated from *trm9*Δ and *trm112*Δ strains were ncm^5^U and ncm^5^s^2^U, respectively (Table 1). We considered the possibility that the ncm^5^ side chain was spontaneously generated from cm^5^ by amidation during the bacterial alkaline phosphatase (BAP) treatment in the digestion step of tRNA to nucleosides for HPLC analysis. To test this hypothesis, synthetic cm^5^U nucleoside was treated in the same way as in the digestion step of tRNA and analyzed by HPLC (Figure 3). We did not detect any conversion of cm^5^U to ncm^5^U (Figure 3) indicating that formation of ncm^5^U is enzymatically catalyzed and not an artifact of the sample preparation procedure.

**Table 1:** Relative amounts of various modified nucleosides in 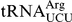 and 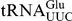 isolated from wild type, *trm9*Δ and *trm112*Δ strains.

	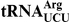			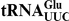					
	cm^5^U/Ψ	ncm^5^U/Ψ	mcm^5^U/Ψ	cm^5^U/Ψ	ncm^5^U/Ψ	mcm^5^U/Ψ	cm^5^s^2^U/Ψ	ncm^5^s^2^U/Ψ	mcm^5^s^2^U/Ψ
WT	0.016	0.044	0.183	ND	ND	ND	0.029	ND	0.220
*trm9*Δ	0.051	0.199	ND	0.065	0.028	ND	0.013	0.191	ND
*trm112*Δ	0.046	0.149	ND	0.044	0.044	ND	0.022	0.161	ND

Pseudouridine (Ψ) was used as the internal control. The numbers displayed are the ratios (modified nucleoside/Ψ). ND: not detected. The modified nucleosides cm^5^U, ncm^5^U, mcm^5^U and Ψ were monitored at 254 nm, and cm^5^s^2^U, ncm^5^s^2^U and mcm^5^s^2^U were monitored at 314 nm as thiolated nucleosides absorb well at this wavelength, while nonthiolated nucleosides do not.

**Figure 4:**
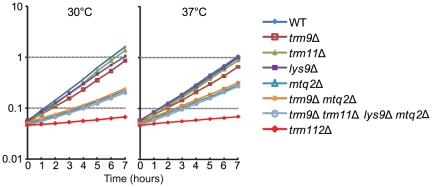
Growth phenotypes. Wild type (UMY2067), *trm112*Δ (UMY3679), *trm9*Δ (UMY3267), *trm11*Δ (UMY3677), *lys9*Δ (UMY3650), *mtq2*Δ (UMY3675), *trm9*Δ *mtq2*Δ (UMY3673) and *trm9*Δ *trm11*Δ *lys9*Δ *mtq2*Δ (UMY3680) strains were cultivated in YEPD at 30°C and 37°C.

In addition to Trm9p, Trm112p also interacts with Trm11p, Lys9p and Mtq2p encoded by non-essential genes, and Ecm16p and Sph1p encoded by essential genes [38], [39], [40], [41]. Therefore, we also analyzed single tRNA species 
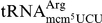
, 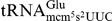 and 
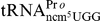
 from *trm11*Δ, *lys9*Δ and *mtq2*Δ strains. Trm11p and Trm112p are essential for formation of the m^2^G nucleoside [42]. Consistently, 
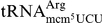
 isolated from *trm11*Δ or *trm112*Δ strains does not have the m^2^G modified nucleoside, whereas the same tRNA from wild-type has m^2^G (Figure 2 and S1). In single tRNAs from *lys9*Δ and *mtq2*Δ strains, there was no notable change in modified nucleosides as assessed by HPLC analysis (Figure S1, data not shown). A deletion of the *TRM112* gene causes a dramatic reduction in growth and a *mtq2*Δ strain also shows a clear reduction in growth, whereas *trm11*Δ, *lys9*Δ or *trm9*Δ strains show mild growth defects in YEPD medium at both 30°C and 37°C (Figure 4). We considered the possibility that strains with multiple null alleles of genes encoding Trm112p interacting proteins would show additive growth defects, possibly mimicking a *trm112*Δ null allele. Since two Trm112p associated proteins, Ecm16 and Sfh1, are encoded by essential genes, we were only able to make strains with combinations of the *trm11*Δ, *lys9*Δ, *trm9*Δ, and *mtq2*Δ alleles. We first made the double mutants *trm11*Δ *lys9*Δ, *trm11*Δ *trm9*Δ, *trm11*Δ *mtq2*Δ, *lys9*Δ *trm9*Δ, *lys9*Δ *mtq2*Δ and *trm9*Δ *mtq2*Δ. No additive growth reduction was observed in any of the constructs at both 30°C and 37°C (Figure 4, data not shown), in contrast to the previously observed growth defect of the *trm9*Δ *mtq2*Δ mutant [33]. Further we made a *trm11*Δ *lys9*Δ *trm9*Δ, *mtq2*Δ quadruple mutant strain that grew like a *mtq2*Δ strain at both 30°C and 37°C (Figure 4). These data show that the poor growth of *trm112*Δ cells is not entirely caused by defects in tRNA modification, eRF1 methylation and dehydrogenase activity in the quadruple mutant. Possibly it is caused by reduced function of Ecm16p or Sfh1p which might require the interaction with Trm112p to be fully active.

### Trm112p/Trm9p complex efficiently incorporates methyl groups into *trm9* substrate tRNA *in vitro*

**Figure 5:**
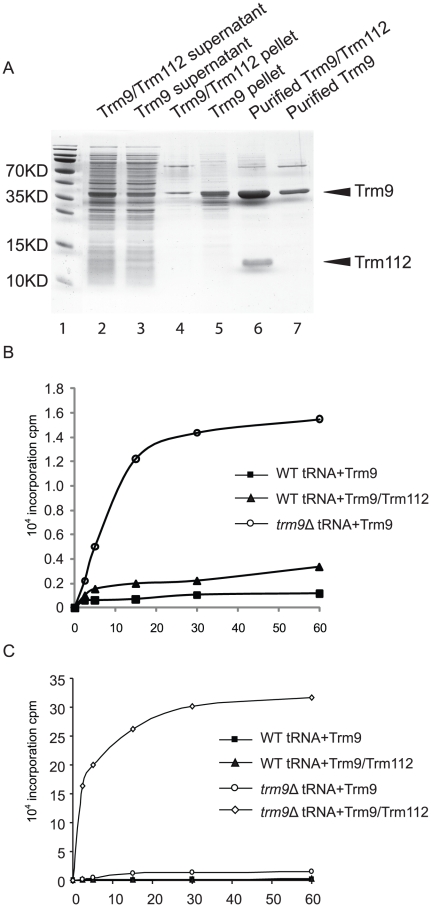
Trm9p/Trm112p complex efficiently catalyzes the methyl incorporation into *trm9* substrate tRNA. (A) SDS-PAGE analysis of histidine tagged Trm9p expressed alone or co-expressed with Trm112p and purified from *E. coli*. The gel was stained with Colloidal Blue (Invitrogen). Lane 1: Molecular weight standard (PageRuler prestained, Fermentas). Lane 2: Soluble fraction of extract from *E. coli* strains expressing Trm112p and histidine tagged Trm9p. Lane 3: Soluble fraction of extract from *E. coli* strains expressing histidine tagged Trm9p. Lane 4: Pellet from crude extract of *E. coli* strains expressing Trm112p and histidine tagged Trm9p. Lane 5: Pellet from crude extract of *E. coli* strains expressing histidine tagged Trm9p. Lane 6: Trm112p co-purified with histidine tagged Trm9 protein. Lane 7: Purified histidine tagged Trm9 protein. (B) [^3^H] methyl incorporation into tRNA as a function of time. Substrates were total tRNA preparations from strain UMY2067 (wild-type) and UMY3267 (*trm9*Δ). (▪) and (▴) are methyl incorporation reactions into wild-type tRNA by using Trm9p or Trm9p/Trm112p as enzyme. (○) is methyl incorporation reaction into *trm9* tRNA by using Trm9p as enzyme. (C). The methyl incorporation into *trm9* tRNA using Trm9p/Trm112p as enzyme (◊), in addition to the reactions in (B).

Trm9p has been shown to catalyze the methyl esterification to mcm^5^U and mcm^5^s^2^U *in vitro* [31]. We cloned the *TRM9* gene into the expression vector pRSF duet to produce 6xHis-Trm9p recombinant protein in *E. coli*. We also made a pRSF duet vector construct, simultaneously expressing the 6xHis-Trm9p recombinant protein and a non-tagged Trm112p. When Trm9p was expressed alone, the majority of Trm9p recombinant protein was insoluble (Figure 5A), and the solubility of Trm9p dramatically improved when Trm112p was co-expressed with Trm9p. Purification of Trm9p by virtue of its 6xHis tag resulted in co-purification of Trm112p (Figure 5A), indicating that Trm9p forms a stable complex with Trm112p.

Purified Trm9p and Trm9/Trm112p complex was used to methylate total tRNA isolated from wild type and a *trm9* deletion strains *in vitro*. Saponification of total tRNA with sodium hydroxide leads to the production of cm^5^U and cm^5^s^2^U from mcm^5^U and mcm^5^s^2^U, and this method has previously been used to generate substrates for Trm9p or ALKBH8 [31], [32]. However, saponification also efficiently degrades tRNA and we found that tRNA isolated from the *trm9* deletion strain was a superior substrate in the methyl esterification assay (data not shown). To track methylation of tRNA substrates *in vitro*, S-adenosylmethionine containing a tritiated methyl donor group was used together with tRNA and purified enzyme. When total tRNA from wild type was used as a substrate, there was a small increase in incorporation of radioactive methyl groups with time using either Trm9p or the Trm9p/Trm112p complex (Figure 5B). In contrast, use of total tRNA from the *trm9*Δ strain and Trm9p leads to a clear but modest increase in the incorporation of radioactive methyl groups (Figure 5B). Moreover, the incorporation of radioactive methyl groups was 20-fold more efficient using Trm9p/Trm112p over Trm9p alone (Figure 5C). Thus, Trm112p is required for Trm9p to methylate its substrate tRNA more efficiently *in vitro* and is a prerequisite *in vivo* as no mcm^5^ nucleosides are formed in a *trm112*Δ mutant (Figure 2, Table 1). In the reaction using tRNA from the *trm9*Δ strain and Trm9p/Trm112p, there was a rapid incorporation of [^3^H] methyl groups in the first 5 minutes that entered to a plateau after 30 minutes (Figure 5C). The reduced incorporation was not due to enzyme inactivation with time as adding more enzyme at 30 minutes did not improve incorporation of radioactivity (data not shown).

**Figure 6:**
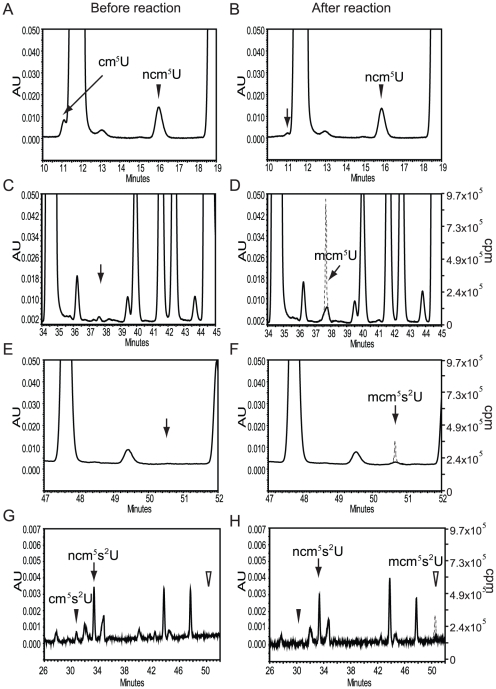
HPLC analysis of total *trm9* tRNA after methyl incorporation by using Trm9p/Trm112p as enzyme. (A–B) Part of the chromatogram between retention time 10 and 19 min is shown. The arrow in B indicates the expected retention time of cm^5^U. (C–D). Part of the chromatogram between retention time 34 and 45 min is shown. The arrow in C indicates the expected retention time of mcm^5^U. (E–F). Part of the chromatogram between retention time 47 and 52 min is shown. The arrow in E indicates the expected retention time of mcm^5^s^2^U. (G–H). Part of the chromatogram between retention time 26 and 52 min is shown. Open and closed arrowheads in G and H indicate the expected retention time of mcm^5^s^2^U and cm^5^U, respectively. Chromatograms in A–F were monitored at 254 nm and at 314 nm in G–H. The dashed line in D, F and H indicates the migration of isotope labeled nucleoside which overlaps with mcm^5^U and mcm^5^s^2^U, respectively. The Y axis to the left corresponds to absorbance units and the Y axis to the right shows the [^3^H] incorporation in cpm.

Based on HPLC analysis, there is an accumulation of cm^5^U, ncm^5^U, and ncm^5^s^2^U in total tRNA from a *trm9*Δ strain compared with a wild-type strain [33] (data not shown). When tRNA isolated from a *trm9*Δ strain was used as substrate *in vitro*, we observed a reduction of the cm^5^U nucleoside and appearance of mcm^5^U (Figure 6A-D, Table 2) consistent with cm^5^U being the substrate of Trm9 [31], [32], [33]. Furthermore, the relative amounts of ncm^5^U and ncm^5^s^2^U did not change after the methylation reaction, showing that these two nucleosides are not substrates of Trm9p/Trm112p under these conditions (Table 2) [33]. By using saponified tRNA, cm^5^s^2^U was suggested to be a substrate for Trm9p or ALKBH8/Trm112 [31], [32]. However, cm^5^s^2^U was not detected in total tRNA isolated from *trm9* or *trm112* mutants [33]. In our analysis of *trm9* total tRNA, we observed a very small peak migrating in the position of cm^5^s^2^U, which was absent after the methylation reaction (Figure 6G-H, Table 2). When [^3^H]-CH_3_ was monitored by flow scintillation analyzer coupled to the HPLC, we found that the incorporated radioactivity migrated with retention times identical to those known for mcm^5^U and mcm^5^s^2^U nucleosides (Figure 6D, F and H). As the signal for the tentative cm^5^s^2^U is very weak, we cannot exclude the possibility that mcm^5^s^2^U originated from another species. These observations are consistent with those shown by Kalhor and Clarke [31], [32] and fully support the assertion that Trm9p is the methyltransferase catalyzing the formation of mcm^5^U from cm^5^U. Why and how ncm^5^U and ncm^5^s^2^U accumulates in tRNAs from strains lacking Trm9p or Trm112p, remains to be elucidated.

**Table 2:** Relative amounts of various modified nucleosides of total tRNA isolated from the *trm9*Δ strain before and after methylation reaction.

	cm^5^U/Ψ	ncm^5^U/Ψ	mcm^5^U/Ψ	cm^5^s^2^U/Ψ	ncm^5^s^2^U/Ψ	mcm^5^s^2^U/Ψ
Before reaction	0.01752	0.04634	ND	0.00081	0.00571	ND
After reaction	0.00227	0.04707	0.01720	ND	0.00521	0.00132

Pseudouridine (Ψ) was used as the internal control. The numbers displayed are the ratios (modified nucleoside/Ψ). ND: not detected. The modified nucleosides cm^5^U, ncm^5^U, mcm^5^U and Ψ were monitored at 254 nm, and cm^5^s^2^U, ncm^5^s^2^U and mcm^5^s^2^U were monitored at 314 nm as thiolated nucleosides absorb well at this wavelength, while nonthiolated nucleosides do not.

### Alternative mechanisms in formation of the mcm^5^ side chain at wobble position

In *trm9*Δ or *trm112*Δ strains, the major species generated are ncm^5^U and ncm^5^s^2^U instead of the expected cm^5^U or cm^5^s^2^U. According to the model proposed in Figure 1, Elongator complex is required for and might directly catalyze the formation of cm^5^U. In the presence of Trm9 and Trm112p, cm^5^U is rapidly converted to mcm^5^U in tRNAs destined to contain a mcm^5^U nucleoside. Those tRNAs destined to contain ncm^5^U are not recognized by Trm9p/Trm112p and ncm^5^U is formed by an uncharacterized enzyme. In order to account for the presence of ncm^5^U and ncm^5^s^2^U in tRNAs that normally should contain mcm^5^U and mcm^5^s^2^U, one has to postulate that in the absence of Trm9p/Trm112p the uncharacterized enzyme responsible for amidation also recognizes these tRNA substrates (Figure 2). For tRNAs that should contain a s^2^ group, the presence of a mcm^5^ side chain has been suggested to be a prerequisite for efficient thiolation [10], [12]. We suggest that the presence of ncm^5^U, but not cm^5^U, in these tRNAs also promotes efficient thiolation, resulting in accumulation of ncm^5^s^2^U (Table 1).

**Figure 7:**
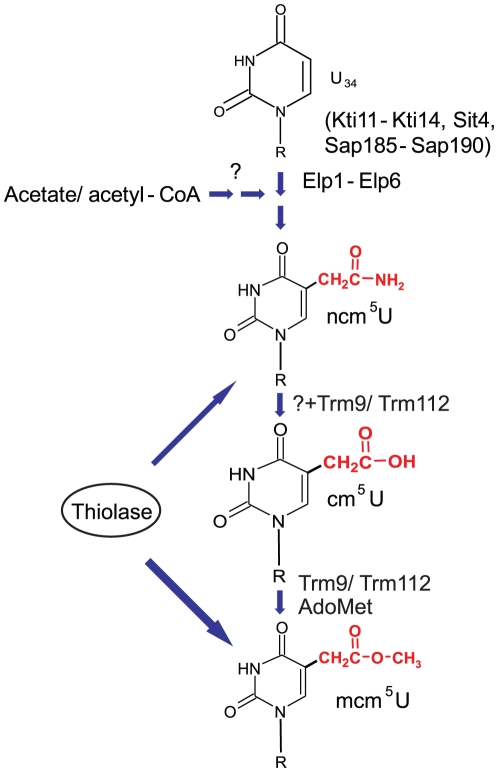
An alternative model for formation of mcm^5^ side chain at wobble uridines. Elongator complex (Elp1-Elp6) and its potential regulators catalyzes the formation of ncm^5^U. The ncm^5^U is converted to cm^5^U by an unknown mechanism in tRNA species that in their mature form should have a mcm^5^ side chain. This unknown mechanism requires Trm9p/Trm112p. In the last step, a methyl group is added to cm^5^U by Trm9p/Trm112p complex in these tRNA species. For tRNAs that should contain a s^2^ group, presence of a mcm^5^ or ncm^5^ side chain is a prerequisite for efficient thiolation.

The observation that the major U_34_ intermediates in 
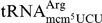
 and 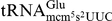 are ncm^5^U and ncm^5^s^2^U in *trm9* and *trm112* mutants also supports an alternative model, i. e. ncm^5^U is generated before cm^5^U (Figure 7). Such a model would require a conversion of ncm^5^U to cm^5^U before the Trm9p/Trm112p complex finally can form mcm^5^U. A similar mechanism has been described in Eubacteria that have mnm^5^ instead of mcm^5^ side chains and the first intermediate in its synthesis is cmnm^5^U [45]. The bi-functional MnmC demodifies cmnm^5^U to nm^5^U and thereafter methylates nm^5^U to form mnm^5^U [45], [46], [47]. By analogy, the Trm9p/Trm112p complex may be involved in two reactions; deamination of ncm^5^U to cm^5^U, and then catalyzing formation of mcm^5^U. The deaminase activity is not necessarily part of Trm9p or Trm112p. In the absence of Trm9p or Trm112p, ncm^5^U accumulates in tRNAs destined to contain mcm^5^s^2^U, like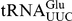. As postulated in model 1, the presence of an ncm^5^ side chain in these tRNAs promotes thiolation, generating ncm^5^s^2^U. MnmC requires flavin adenine dinucleotide (FAD) as co-factor in the de-modification reaction and SAM in the methylation reaction. We performed an *in vitro* reaction with [^3^H]AdoMet in the presence or absence of FAD. We assumed if ncm^5^U is converted to cm^5^U in the presence of FAD, more [^3^H]-methyl groups would be incorporated into total tRNA isolated from *trm9* deletion strain when FAD is included in the reaction. Reactions conducted in the presence of FAD did not increase the incorporation of [^3^H]-methyl into *trm9* deletion tRNA, nor did it decrease the overall amount of ncm^5^U as analyzed by HPLC (data not shown). We also investigated the potential use of other cofactors in the conversion of ncm^5^U to cm^5^U such as NAD^+^ and NADP^+^ without success (data not shown). It remains to be elucidated which of these two alternative pathways for formation of mcm^5^ side chains is used.

## Supporting Information

Figure S1
**HPLC analysis of modified nucleosides in** 
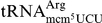
 **isolated from wild-type,** ***trm11***Δ**,** ***lys9***Δ **and** ***mtq2***Δ **strains.** Arrows in red and black indicate expected retention time of mcm^5^U and cm^5^U, respectively. Arrow heads in red and black indicate expected retention time of m^2^G and ncm^5^U, respectively. (A–D), Part of the chromatogram between retention times 34 and 44 min is shown. (B), Part of the chromatogram between retention times 7 and 17 min is shown. The small peak in wild-type at 14 min represents an unrelated compound with a spectrum different from ncm^5^U. Absorbance at 254 nm (AU) was used to create the chromatograms.(EPS)

Table S1 Strains and primers used in this study (see also [48] and [49]).
(DOC)
