# Supplementary material for: Unexpected Accumulation of ncm5U and ncm5s2U in a trm9 Mutant Suggests an Additional Step in the Synthesis of mcm5U and mcm5s2U
Source: PLoS One. 2011 Jun 7;6(6):e20783. doi: 10.1371/journal.pone.0020783 (PMC3110198; doi:10.1371/journal.pone.0020783)
Supplement: Table S1 — Strains and primers used in this study (see also [48] and [49]). (DOC) [file pone.0020783.s002.doc]

Table 1.

A. Strains used in this study

| Yeast strains | Genotype | Source |
| --- | --- | --- |
| W303-1A | *MATa leu2-3,112 trp1-1 can1-100 ura3-1 ade2-1 his3-11,15* | [48] |
| W303-1B | *MATα leu2-3,112 trp1-1 can1-100 ura3-1 ade2-1 his3-11,15* | [48] |
| UMY3104 | *MATa/MATα leu2-3,112/ leu2-3,112 trp1-1/ trp1-1 can1-100/ can1-100 ura3-1/ ura3-1 ade2-1/ ade2-1 his3-11,15/ his3-11,15* | [6] |
| UMY3169 | *MATa/MATα his3Δ1 / his3Δ1 leu2Δ0 / leu2Δ0 ura3Δ0 /ura3Δ0 met15Δ0 /MET15 LYS2/ lys2Δ0* | This study |
| UMY3267 | *MATa leu2-3,112 trp1-1 can1-100 ura3-1 ade2-1 his3-11,15 trm9::KanMX* | [49] |
| UMY3297 | *MATα leu2-3,112 trp1-1 can1-100 ura3-1 ade2-1 his3-11,15 trm9::KanMX* | [49] |
| UMY3330 | *MATa ura3-1 leu2-3,112 trp1-1 his3-11,15 can1-100 ade2-1 SSD1-v1 SUP4 trm112::KanMX6* | This study |
| UMY3650 | *MATa leu2-3,112 trp1-1 can1-100 ura3-1 ade2-1 his3-11,15 lys9::KanMX* | This study |
| UMY3651 | *MATα leu2-3,112 trp1-1 can1-100 ura3-1 ade2-1 his3-11,15 lys9::KanMX* | This study |
| UMY3663 | *MATa leu2-3,112 trp1-1 can1-100 ura3-1 ade2-1 his3-11,15 trm11::KanMX lys9::KanMX* | This study |
| UMY3665 | *MATa leu2-3,112 trp1-1 can1-100 ura3-1 ade2-1 his3-11,15 trm9::KanMX lys9::KanMX* | This study |
| UMY3667 | *MATa leu2-3,112 trp1-1 can1-100 ura3-1 ade2-1 his3-11,15 trm9::KanMX trm11::KanMX* | This study |
| UMY3669 | *MATa leu2-3,112 trp1-1 can1-100 ura3-1 ade2-1 his3-11,15 mtq2::KanMX trm11::KanMX* | This study |
| UMY3671 | *MATa leu2-3,112 trp1-1 can1-100 ura3-1 ade2-1 his3-11,15 mtq2::KanMX lys9::KanMX* | This study |
| UMY3673 | *MATa leu2-3,112 trp1-1 can1-100 ura3-1 ade2-1 his3-11,15 mtq2::KanMX trm9::KanMX* | This study |
| UMY3675 | *MATa leu2-3,112 trp1-1 can1-100 ura3-1 ade2-1 his3-11,15 mtq2::KanMX* | This study |
| UMY3676 | *MATα leu2-3,112 trp1-1 can1-100 ura3-1 ade2-1 his3-11,15 mtq2::KanMX* | This study |
| UMY3677 | *MATa leu2-3,112 trp1-1 can1-100 ura3-1 ade2-1 his3-11,15 trm11::KanMX* | This study |
| UMY3678 | *MATα leu2-3,112 trp1-1 can1-100 ura3-1 ade2-1 his3-11,15 trm11::KanMX* | This study |
| UMY3679 | *MATa leu2-3,112 trp1-1 can1-100 ura3-1 ade2-1 his3-11,15 trm112::KanMX* | This study |
| UMY3680 | *MATα leu2-3,112 trp1-1 can1-100 ura3-1 ade2-1 his3-11,15 trm112::KanMX* | This study |
| UMY3680 | *MATa leu2-3,112 trp1-1 can1-100 ura3-1 ade2-1 his3-11,15 trm9::KanMX trm11::KanMX lys9::KanMX mtq2::KanMX* | This study |

B. Primers used in this study

| Name | Number | Oligo |
| --- | --- | --- |
| *MTQ2* deletion | 2104 | 5’-AAATATAATATTGATAAACTTAACACAGGGTGAGA AAGGTGAAGTCGGATCCCCGGGTTAATTAA-3’ |
|  | 2105 | 5’-TAAAGACACAGGTTATCAATTATAACGTGAAAGGTT TTGCAACTGGAATTCGAGCTCGTTTAAAC-3’ |
| *TRM112* deletion | 1392 | 5’-AGTTGTCTTTTCGTCTTGCGTGCCCACACACAGAGAT CTCGCTTGATCGGATCCCCGGGTTAATTAA-3’ |
|  | 1391 | 5’-CCTATGATCTCTTCGGCTCTACACATCATATTACTAG CCTAGTCAACGAATTCGAGCTCGTTTAAAC-3’ |
| *TRM9* deletion | 1035 | 5’-GTCCTCACTGATGACTTGGTC-3’ |
|  | 1036 | 5’-GGGTGTAGACGTTTGAGTGG-3’ |
| *TRM11* deletion | 1950 | 5’-GTCGAATCGTTTGAAGGGGC-3’ |
|  | 1951 | 5’-CTTCTGCGCAAGATCTTCAAG-3’ |
| *LYS9* deletion | 2059 | 5’-GATTGCGGTTACTACTGACC-3’ |
|  | 2060 | 5’-AGGTAGACGACTTACTAGCG-3’ |
| *TRM9* clone | 2015 | 5’-GCGGATCCGAGATAAACCAAGCGGCTG-3’ |
|  | 2016 | 5’-GCAAGCTTTCATCTCTTCTGGGCCACC-3’ |
| *TRM112* clone | 2013 | 5’-GCCATATGAAGTTCTTAACCACCAACTTC-3’ |
|  | 2014 | 5’-GCCTCGAGTTATACCAGGTGTGGAGGTAAC-3’ |
